# Effects of Multimedia Assisted Song Integrated Teaching on College Students' English Learning Interests and Learning Outcomes

**DOI:** 10.3389/fpsyg.2022.912789

**Published:** 2022-06-21

**Authors:** Yu-Zhou Luo, Xiang-Yang Kong, Yu-Yang Ma

**Affiliations:** Business School, Guilin University of Technology, Guilin, China

**Keywords:** multimedia assisted, English teaching, learning interest, enterprise, learning outcome

## Abstract

In the process of globalization, the English language not only represents British and American culture, but it has also gradually become a language used all over the world, and it has become essential for many people to learn it as a second language. Education is the century business of a nation. At the same time, to meet the needs of E generation, I generation, and touch-screen generation students, teachers are increasingly undertaking multimedia-integrated curriculum design and instruction. Teachers are no longer knowledge providers, but they are expected to provide students with a personalized learning model and guide and support them in a timely manner. This study included a sample of business students from Guilin University of technology. A total of 216 students participated in a 16-week (3 h per week, a total of 48 h) course of experimental teaching. The research results showed that 1. multimedia assisted, song integrated English teaching affected learning interest, 2. That multimedia assisted, song integrated English teaching affected learning outcomes, and 3. That learning interest had significantly positive effects on learning outcome. Based on these results, this study contributes to improving college students' English listening, speaking, reading, and writing skills via multimedia teaching, which also facilitated their interest and ability to achieve the learning outcomes.

## Introduction

English is now the predominant common language for global commerce, academic research or conferences, political exchange, and information communication (Meyers et al., 2019). More than 80% of websites in the world use English as the language of communication (D'Amico, 2018), and more than one billion people use English to communicate (Pham and Huynh, 2018). It is a fact that English is the global lingua franca. In the process of globalization, English not only represents British and American culture but has also gradually become a language widely used all over the world, meaning it is unavoidable, and that it is essential for many people to learn it as a second language.

Today's education system works with digitally native students who have grown up in an environment with various digital products. From birth, they have encountered televisions, video games, and the Internet, and have adopted the mindset and information acquisition of older generations, especially the styles of information communication they involve. Having grown up in this digital nation, they love motion videos, graphic explanations, and the Internet, and constantly use digital products, meaning they worry about battery life and charging. Educators face attention deficits and distractions, which are common problems today. and should actively look for causes and find appropriate teaching models to help them improve their learning styles and attitudes.

Education is the business of the century. To cope with the E generation, I generation, and touch-screen generation students, increasing numbers of teachers have integrated multimedia tools into curriculum design and instruction. In the context of this student-centered instruction, tenacious flexibility is one of the most required qualities in teachers. Teachers are no longer just knowledge providers, they are expected to provide students with a personalized learning model and guide and support them in a timely manner. As students are placed at the center of learning, teachers must become good managers of the learning environment and creative curriculum designers. It is normal for modern people to hold a device in their hands, and instead of banning its use, this strong attraction can be transformed into a learning tool. Disseminated via these digital channels, the pop music industry is popular worldwide. Through multimedia information transfer, students have heard English pop songs or watched nusic videos. Many songs reflect the spirit of young students, so they can be more easily reconciled.

This study contributes to improving college students' English listening, speaking, reading, and writing skills via multimedia teaching. The students' English learning interests and learning outcomes can be facilitated through multimedia teaching.

## Literature Review and Hypothesis

English songs are integrated into multimedia instruction because English songs are a combination of music and simple language. In addition, they have a sense of rhythm. They have interesting subjects and are rich in meaning. These features are excellent for remedial teaching. Frequent singing can be used as pronunciation practice for children and can cultivate the temperament of language beginners (Pavia et al., 2019). The clear, easy-to-understand, harmonic, and easy-to-sing lyrics are a short rhythmic literary composition (Arigita-García et al., 2021). Integrating English songs into the classroom can fully develop the feeling that music is relaxing and has catchy characteristics that promote learning motivation. Relevant research has indicated that English songs, with their phonology and syllable structure, are appropriate materials for exploring language (Chen, 2018). Hong et al. (2020) mentioned that, unlike traditional English pop song teaching, which simply played music, multimedia assisted popular English teaching is a combination of music and image to promote the learning ability of learners' left and right hemispheres and enable learners to engage in the lyrics and songs. The multimedia assisted songs can successfully attract students' attention, increase learning interest, create a relaxed and pleasant learning environment, and reduce learning anxiety. With repeated listening, the integrated English curricula can increase the confidence of students as they often can sing English songs regardless of academic achievement levels. Dingyloudi et al. (2019) stated that there are many themes in western pop songs that attract young students, such as the subculture of friendship, loneliness, adventure, love, imagination, fashion, and campus. Appreciating such multimedia assisted songs can better arouse learning interest in consonance. Megreya et al. (2021) pointed out that a large number of contemporary popular slogans in English pop songs used media assistance to support avant-garde images for learning English vocabulary not found in textbooks, but that they mainly served to arouse students' learning interests. Therefore, the following hypothesis is proposed in this study:

**H1**: Multimedia assisted, song integrated English teaching has an effect on learning interest.

English songs are the best tools for learning English. Music can help improve temperament, express emotions, and effectively relieve learners' fear of a second language. When the mood is good and cheerful, learning will naturally be fun (Ferawaty, 2018). If teachers can create a happy English learning environment and relax tense emotions through favorite English pop songs, both students and teachers will enjoy it. Thus, learning is a double outcome with half the effort and a win-win situation for both teachers and students (Brandmo and Bråten, 2018). Teaching through English songs can help learners understand western music culture and the background of singers and learn the vocabulary, phrases, and grammatical structure of song lyrics. Integrated music teaching can promote learners' English listening, speaking, reading, and writing skills (Hwang et al., 2020).

Hwang et al. (2019) believe that the instructional, entertainment, musical, and cultural functions of multimedia assisted English pop song teaching lead to the appreciation of English pop songs. Multimedia assistance serves to activate otherwise boring teaching and involve English learning in authentic life to improve students' English learning outcomes. Pulasthi and Gunawardhana (2021) mentioned that when listening to multimedia assisted songs, learners were relaxed and happy and awaited the multimedia assisted songs with a curious attitude. In such a learning environment, learners increased their confidence and performance in language learning, because, with musical accompaniment, all learners opened their mouths to sing to promote the learning outcome. Adkins-Jablonsky et al. (2021) indicated that teaching with English pop songs can improve students' English. English pronunciation is different from Chinese pronunciation, and the stress, intonation, accent, and rhythm of English words and sentences often cause difficulties for some learners. Singing English songs enabled students to improve their oral skills and listening comprehension, and the use of multimedia assisted English pop song teaching enabled students to engage in the learning process to promote learning outcomes. Accordingly, this study hypothesizes the following.

**H2**: The multimedia assisted English pop song integrated teaching has a positive effect on learning outcome.

Learning interest, an important factor in student learning, can affect attention to the subject, goal setting, self-adjustment, use of cognitive strategies, and learning performance. In addition, interest is closely related to willingness, effort, and persistence in selecting homogeneous content for the next learning activity (Munavvar and Fazila, 2018). In practice, teachers discover that learning interest enables students to enjoy learning (Loingsigh and Mozzon-McPherson, 2020). In this respect, students with high learning interest show better self-directed learning compared to students with low learning interest, and they do not easily give up when they encounter difficulties (Jiang and Zhang, 2020). Wang et al. (2018) regarded learning interest as the time and effort students are willing to invest in the tasks. Students with stronger learning interest, higher activity, higher achievement motivation, and a positive attitude toward the completion of tasks can present higher confidence in competing matters. Attitudes toward task completion revealed that students' interest in the task resulted in higher learning outcomes. Psychologists emphasize that it is important to arouse learners' interest in learning materials to improve learning outcomes (Baydas and Cicek, 2019). If teachers succeed in arousing students' learning interest in the learning process and transforming students from passive recipients to active learners, the learning activity has a positive impact on both teachers and learners (Verkijika and De Wet, 2019). Learning interest was closely related to learning outcome; learning interest can facilitate the perception of learning tasks, guide meaningful learning, and provide further motivation to learn (Hollingsworth et al., 2018). Therefore, the following hypothesis is proposed in this study.

**H3**: Learning interest has a significant positive effect on learning outcomes.

## Methodology

### Measurement of Research Variables

#### Learning Interest

Following Hong et al. (2021), learning interest includes two dimensions.

1. Personal interests: the relatively persistent characteristic of certain matters that cause individuals to continuously engage in the activity.
2. Situational interests: situational interest refers to interests that are triggered by specific matters or conditions in the environment, e.g., movies, or topic content.

#### Learning Outcome

Following Kang et al. (2019), the dimensions are described as follows.

1. The learning effect: this dimension includes test performance, processing time, and semester performance.
2. The learning gain: this dimension includes learning satisfaction, achievement, and preference.

### Research Object and Sampling Data

This study included business students at Guilin University of Technology. A total of 216 students participated in a 16-week (3 h per week, a total of 48 h) experimental teaching. Data from the questionnaire were analyzed using SPSS. Factor analysis, reliability analysis, regression analysis, and analysis of variance were used to test the hypotheses.

### Analysis Method

Analysis of variance was used to discuss the difference between multimedia assisted and song integrated English teaching in terms of learning interest and learning outcome. Similarly, regression analysis was applied to understand the relationship between learning interest and learning outcome.

## Results

### Reliability and Validity Analysis

As a result of the factor analysis, two factors of learning interest were extracted as “personal interests” (eigenvalue = 3.182, α = 0.90) and “situational interests” (eigenvalue = 2.734, α = 0.88). The cumulative covariance explained was calculated as 78.473%.

Additionally, two factors were extracted under the construct of learning outcome through factor analysis. These two factors are “learning effect” (eigenvalue = 2.529, α = 0.91) and “learning gain” (eigenvalue = 2.241, α = 0.93). The cumulative covariance explained was found as 82.366%.

### Effects of Multimedia Assisted, and Song Integrated English Teaching on Learning Interest and Learning Outcome

#### Difference Analysis of Multimedia Assisted, and Song Integrated English Teaching in Learning Interest

The analysis of variance, which discusses the difference between multimedia assisted song integrated English teaching and learning interest was conducted. According to the analysis, significant differences were found between personal interests before and after multimedia assisted song integrated English teaching (Table 1). It was found higher after teaching (4.06) compared to the level before teaching (3.73). Situational interests also revealed significant differences in before and after multimedia assisted song integrated English teaching. The level was found to be higher after teaching (4.11) than before (3.66). In this case, H1 was supported.

**Table 1:** Difference analysis of multimedia assisted song integrated English teaching in terms of learning interest.

**Variable**		**F**	***P***	**Scheffe *post hoc***
Multimedia assisted song Integrated English teaching	Personal interests	13.432	0.000*	After teaching (4.06) > before teaching (3.73)
	Situational interests	22.133	0.000*	After teaching (4.11) > before teaching (3.66)

^*^p <0.05.

#### Difference Analysis of Multimedia Assisted Song Integrated English Teaching in Terms of Learning Outcome

The analysis of variance, which discusses the difference between multimedia assisted song integrated English teaching in terms of learning outcome was used. According to the results, the learning effect showed a significant difference before and after multimedia assisted song integrated English teaching (Table 2). The effect on learning was higher after teaching (4.17) than before (3.84). Similarly, learning gain was found to be higher after teaching (4.26) compared to before teaching (3.88). Consequently, H2 was supported.

**Table 2:** Difference analysis of multimedia assisted English song integrated English teaching in learning outcome.

**Variable**		**F**	***P***	**Scheffe post hoc**
Multimedia assisted song Integrated English teaching	Learning effect	18.442	0.000*	After teaching (4.17) > before teaching (3.84)
	Learning gain	27.592	0.000*	After teaching (4.26) > before teaching (3.88)

*p <0.05.

### Correlation Analysis of Learning Interest and Learning Outcome

#### Correlation Analysis of Learning Interest and Learning Effect

A correlation analysis was conducted to test H3. The results of the analysis revealed significant effects of personal interests (β = 2.096^**^) and situational interests (β = 2.185^**^) on the learning effect (Table 3).

**Table 3:** Analysis of the effect of learning interest on learning outcome.

**Dependent variable →**	**Learning outcome**			
**Independent variable↓**	**Learning effect**		**Learning gain**	
**Learning interest**	**β**	***P***	**β**	***P***
Personal interests	2.096**	0.000	2.216**	0.000
Situational interests	2.185**	0.000	2.375**	0.000
F	25.637		32.546	
Significance	0.000***		0.000***	
R^2^	0.243		0.307	
adjusted R^2^	0.221		0.286	

*p <0.05 and **p <0.01.

*Data source: Self-organized in this study*.

#### Correlation Analysis of Learning Interest and Learning Gain

The correlation analysis conducted on H3 revealed the significant effects of personal interests (β = 2.216^**^) and situational interests (β = 2.375^**^) on learning gain. Therefore, H3 was supported.

## Discussion

In this study, Multimedia-based English teaching promotes interest in English learning and increases learning outcomes. Teachers should not simply provide a large amount of teaching materials, they should provide systematically plan multimedia teaching content and instructional strategies (Verkijika and De Wet, 2019). Students' prerequisite abilities should also be evaluated and then appropriate multimedia materials should be provided according to their abilities because students without sufficient prior knowledge cannot easily construct solid new knowledge. In this respect, teachers should also consider the integration of other multimedia design strategies when using interactive multimedia materials in the classroom. In multimedia-supported English classes, various multimedia instructions aligned with the course content can be used to promote students' learning interests. Schools provide a method to effectively improve student learning outcomes to more easily achieve instructional objectives and enhance students' international competitiveness. The application of multimedia assisted English song integrated English teaching is learner-centered, and technology-based. Aligning multimedia-assisted English teaching with instructional design enables richer and more diverse instructional methods in learning areas and enhances students' attention, memory, and active learning to effectively promote overall learning outcomes.

Heterogeneous grouping was done according to English proficiency before multimedia assisted song integrated English teaching. Each group contained 4–5 students. In order to test learning outcomes, group evaluation was adopted. The group with the highest score won the game. Besides, the regular order and participation of the groups were also evaluated. Appropriate rewards were used to increase each student's participation and cooperation. After the groups completed the learning process, teachers held singing contests for students in which they prepared a show after classes. The inclusion of props and dance could promote students' English potential. Multimedia assisted song integrated English teaching relies heavily on multimedia equipment available to schools to improve the environment of the e-classroom and enhance the convenience of use, so that teachers and students enjoy using it and teaching becomes more effective. Moreover, schools can budget for purchasing Bluetooth microphones that students can connect to the Bluetooth in their mobile phones during multimedia assisted song integrated English teaching. Sound effects of a professional microphone can boost students' confidence in singing. Facing the students in E-generation, I-generation, and touch-screen generation, teachers must be prepared to change teaching methods if they require students to change their learning attitudes and methods. Scholls can organize workshops on multimedia assisted teaching and invite teachers and scholars with experience of multimedia assisted teaching to demonstrate and share insights, providing teachers with more opportunities for creativity in multimedia assisted English teaching and promoting teachers' interests in and competencies when using multimedia assistance. With constant self-realization and the accumulation of experience, teachers can use multimedia assisted teaching and make teaching more attractive. Moreover, schools can organize activities such as a school English song contest, English choir contest, or I am MV director, encouraging student participation and creating a campus environment with English awareness. Teachers can build personal YouTube channels to teach English and students can subscribe so the channel, meaning English learning is not restricted to the school environment. Teachers can include selected MVs that are for students' learning or they can record instructional videos and open a comments section for feedback, as if they are popular among students they will promote learning interests and learning outcomes.

## Conclusion

This study shows that multimedia assisted song integrated English teaching promotes students' interest in learning English. Traditional classroom teaching is limited to place, time, resources, and materials, and teachers might only use blackboards/whiteboards and textbooks for teaching. They also often ignore more learner-centered approaches and do not care about the learning interests of students (Williams et al., 2019).

Based on learners, this study explored the use of popular English songs in multimedia assisted integrated teaching. During the process of multimedia assisted English song playing, the audio-visual stimuli facilitated a relaxed and pleasant mood in students, enabling them to enjoy classes and encouraging them to learn actively and sing popular English songs, and even relax their limbs and sway with the music. Students participated in singing English songs and were deeply touched (Yang and Quadir, 2018).

In terms of learning outcomes, students confirmed that multimedia assisted song integrated English teaching improved their English listening, speaking, reading, and writing skills. English skills and learning outcomes can be promoted by providing complete song lyrics in class, coordinating the schedule of semester tests, and extracting key vocabulary, phrases, and grammatical structures. Students gained comprehensive practice through the use of multimedia assisted song integrated English teaching.

## Data Availability Statement

The original contributions presented in the study are included in the article/supplementary material, further inquiries can be directed to the corresponding author.

## Author Contributions

Y-ZL performed the initial analyzes and wrote the manuscript. X-YK and Y-YM assisted in the data collection and data analysis. All authors revised and approved the submitted version of the manuscript.

## Conflict of Interest

The authors declare that the research was conducted in the absence of any commercial or financial relationships that could be construed as a potential conflict of interest.

## Publisher's Note

All claims expressed in this article are solely those of the authors and do not necessarily represent those of their affiliated organizations, or those of the publisher, the editors and the reviewers. Any product that may be evaluated in this article, or claim that may be made by its manufacturer, is not guaranteed or endorsed by the publisher.
